# Pre-CCRT 18-fluorodeoxyglucose PET-CT improves survival in patients with advanced stages p16-negative oropharyngeal squamous cell carcinoma via accurate radiation treatment planning

**DOI:** 10.1186/s40463-023-00623-y

**Published:** 2023-02-13

**Authors:** Tsung-Ming Chen, Wan-Ming Chen, Mingchih Chen, Ben-Chang Shia, Szu-Yuan Wu

**Affiliations:** 1 Department of Otolaryngology-Head and Neck Surgery, Shuang Ho Hospital, Taipei Medical University, Taipei, Taiwan; 2 Graduate Institute of Business Administration, College of Management, Fu Jen Catholic University, Taipei, Taiwan; 3 Artificial Intelligence Development Center, Fu Jen Catholic University, Taipei, Taiwan; 4 Department of Food Nutrition and Health Biotechnology, College of Medical and Health Science, Asia University, Taichung, Taiwan; 5 Division of Radiation Oncology, Lo-Hsu Medical Foundation, Lotung Poh-Ai Hospital, No. 83, Nanchang St., Luodong Township, Yilan County 265 Taiwan; 6 Big Data Center, Lo-Hsu Medical Foundation, Lotung Poh-Ai Hospital, Yilan, Taiwan; 7 Department of Healthcare Administration, College of Medical and Health Science, Asia University, Taichung, Taiwan; 8 Cancer Center, Lo-Hsu Medical Foundation, Lotung Poh-Ai Hospital, Yilan, Taiwan; 9 Centers for Regional Anesthesia and Pain Medicine, Taipei Municipal Wan Fang Hospital, Taipei Medical University, Taipei, Taiwan; 10 Department of Management, College of Management, Fo Guang University, Yilan, Taiwan

**Keywords:** Concurrent chemoradiotherapy, ^18^FDG PET–CT, OPSCC, Survival, Clinical stages

## Abstract

**Purpose:**

No large-scale prospective randomized study with a long-term follow-up period has evaluated the survival outcomes of preconcurrent chemoradiotherapy (CCRT) 18-fluorodeoxyglucose positron emission tomography–computed tomography (^18^FDG PET–CT) in patients with non–human papillomavirus (HPV)-associated oropharyngeal squamous cell carcinoma (OPSCC).

**Patients and Methods:**

We included patients with stage I–IVA p16-negative OPSCC receiving definitive CCRT and categorized them into two groups according to pre-CCRT ^18^FDG PET–CT and compared their outcomes: the case group consisted of patients who underwent pre-CCRT ^18^FDG PET–CT, whereas the comparison group consisted of patients who did not receive pre-CCRT ^18^FDG PET–CT.

**Results:**

The final cohort consisted of 3942 patients (1663 and 2279 in the case and comparison groups, respectively). According to multivariable Cox regression analysis, pre-CCRT ^18^FDG PET–CT was not a significant prognostic factor for overall survival in patients with stages I–II of p16-negative OPSCC receiving standard CCRT. The adjusted hazard ratio (95% confidence interval) of all-cause death for the patients with advanced stages (III–IVA) of p16-negative OPSCC receiving pre-CCRT ^18^FDG PET–CT was 0.75 (0.87–0.94, *P* = 0.0236).

**Conclusions:**

Routine use of pre-CCRT ^18^FDG PET–CT is not necessary for each patient with p16-negative OPSCC. Pre-CCRT ^18^FDG PET–CT is associated with improved survival in patients with stage III–IVA p16-negative OSCC, but might be not in those with stage I–II p16-negative OPSCC.

**Condensed abstract:**

No large-scale prospective randomized study with a long-term follow-up period has evaluated the survival outcomes of preconcurrent chemoradiotherapy (CCRT) 18-fluorodeoxyglucose positron emission tomography–computed tomography (^18^FDG PET–CT) in patients with p16-negative oropharyngeal squamous cell carcinoma (OPSCC). Our study is the first, largest, homogenous modality study on PET–CT including a long-term follow-up cohort to examine the survival outcomes of pre-CCRT ^18^FDG PET–CT or non-pre-CCRT PET–CT for patients with p16-negative OPSCC receiving standard CCRT stratified by different clinical stages. Routine use of pre-CCRT ^18^FDG PET–CT is not necessary for each patient with p16-negative OPSCC. Pre-CCRT ^18^FDG PET–CT is associated with improved survival in patients with stage III–IVA p16-negative OPSCC, but might be not in those with stage I–II p16-negative OPSCC.

## Introduction

Oropharyngeal squamous cell carcinoma (OPSCC) is a relatively uncommon malignancy, with approximately 123,000 cases of oropharyngeal and hypopharyngeal cancer being diagnosed and approximately 79,000 deaths occurring worldwide each year [1]. In Taiwan, OPSCC is the third leading form of head and neck cancer and the third leading cause of head and neck cancer–related deaths [2]. Human papillomavirus (HPV) infection is associated with the development of OPSCC [3]. Biomarkers commonly used in clinical practice include p16 expression (determined through immunohistochemistry) and HPV 16 viral load (detected through real-time polymerase chain reaction) [3,4]. Patients with p16-positive OPSCC as HPV-associated OPSCC typically have more favorable prognosis than do those with p16-negative (non-HPV-associated) OPSCC [5]. Thus, improving the overall survival (OS) of patients with p16-negative OPSCC has become increasingly crucial because of its poorer survival outcomes compared with p16-positive OPSCC [5,6].

18-Fluorodeoxyglucose (^18^FDG) positron emission tomography (PET) and integrated ^18^FDG PET–computed tomography (^18^FDG PET–CT) have replaced other modalities for the detection of distant metastases and synchronous second primary tumors [7,8]. However, false-positive findings are common, highlighting the need to histologically confirm any sites of abnormal uptake [9,10]. ^18^FDG PET–CT is sensitive and superior for the evaluation of deep lesions, whereas panendoscopy is highly accurate for the evaluation of smaller or more superficial second primary mucosal lesions [9,11,12]. Therefore, ^18^FDG PET–CT and direct mucosal inspection play crucial complimentary roles in the diagnosis of head and neck cancers [13].

Early-stage OPSCC can be treated with either primary surgery or definitive radiotherapy (RT) plus chemotherapy or not as a therapeutic modality [14,15]. Definitive RT and primary surgery yield similar rates of local control and survival for early-stage OPSCC [15]. According to the National Comprehensive Cancer Network (NCCN) guidelines (Category 2B), some physicians recommend concurrent RT (CCRT) for patients with early-stage OPSCC [14]. Intensity-modulated RT (IMRT) to the primary tumor and regional lymph nodes is the optimal RT technique [14]. Functional organ preservation approaches utilizing the combination of chemotherapy and RT, that is, CCRT, without surgery are more commonly used for advanced stages of OPSCC [16,17]. Imaging studies (computed tomography [CT], magnetic resonance imaging [MRI], PET, and integrated PET–CT) are crucial to assess the degree of local infiltration, the involvement of regional lymph nodes, and the presence of distant metastases or second primary tumors [13,18,19]. The evaluation of regional lymph nodes has considerably improved with the development of imaging modalities such as integrated PET–CT [20].

The role of a routine ^18^FDG PET–CT scan in the staging of patients with p16-negative OPSCC remains unclear. ^18^FDG PET–CT imaging is indicated for patients with a high risk of metastatic disease, those with equivocal findings on CT or MRI, and those with an increased risk of a second malignancy who would not be undergoing panendoscopy (laryngoscopy, esophagoscopy, or bronchoscopy) [5,10,13,19]. Furthermore, ^18^FDG PET–CT is beneficial for the restaging of head and neck cancer after initial therapy [21,22]. However, no comparative study with a long-term follow-up has examined the survival benefits of pretreatment ^18^FDG PET–CT in patients with p16-negative OPSCC receiving CCRT. Therefore, this large-scale retrospective cohort study investigated the benefits of pretreatment ^18^FDG PET–CT in patients with p16-negative OPSCC.

## Patients and methods

### Data source and study cohort

From the Taiwan Cancer Registry Database (TCRD), we enrolled patients who had received a diagnosis of p16-negative OPSCC between January 1, 2008, and December 31, 2018. The follow-up duration was from the index date to December 31, 2019. Biomarkers commonly used in clinical practice include p16 expression (determined through immunohistochemistry) and HPV 16 viral load (detected through real-time polymerase chain reaction) [3,4]. Either the HPV 16 viral load or p16 expression status can be used as a marker of HPV infection depending on the institution [23]. Therefore, in our study, p16-negative OPSCC was defined as the absence of p16 expression. The study protocols were reviewed and approved by the Institutional Review Board of Tzu-Chi Medical Foundation (IRB109-015-B). The cancer registry database of the Collaboration Center of Health Information Application contains detailed cancer-related information regarding clinical stages, pathological types, RT doses, RT techniques, and CT regimens used [24–26]. In this study, the diagnoses of enrolled patients were confirmed according to their pathological data and p16 expression status. Patients who had received a diagnosis of OPSCC were confirmed to have no other cancer or distant metastasis.

### Selection of cases and controls

Inclusion criteria were having a diagnosis of p16-negative OPSCC, being aged > 20 years, and having American Joint Committee on Cancer (AJCC) clinical stage I–IVA cancer without metastasis. The AJCC 8^th^ edition was used for staging cancer in all patients. Exclusion criteria were having a history of cancer before the diagnosis of OPSCC, metastasis, missing sex data, in situ carcinoma, and nonsquamous cell carcinoma and being aged < 20 years. The index date was the date on which patients received CCRT. In addition, we excluded patients with OPSCC who did not receive any treatment, did not receive concurrent chemotherapy with at least two agents containing platinum [27], did not receive RT with IMRT, did not complete the RT course (< 70 Gy), did not begin standard CCRT within 3 months after diagnosis, or did not receive CCRT (sequential CT and RT). Standard CCRT comprises concurrent chemotherapy with two agents containing platinum and IMRT at a total dose of 70 Gy in daily fractions. All included patients received standard CCRT. Highly conformal external beam RT techniques (such as IMRT) and its iteration (volumetric modulated arc therapy) were allowed in this study. Only 1.63% and 1.77% of patients who received pre-CCRT PET–CT and non-pre-CCRT PET–CT with IMRT, respectively, did not complete the RT course; we excluded these patients. No significant difference in the completion rate of the RT course was observed between the case and control groups. Patients who received stereotactic boost were not included in this study. We included only patients with OPSCC who underwent pre-CCRT PET–CT or non-pre-CCRT PET–CT with IMRT, but not neoadjuvant or adjuvant chemotherapy. In this study, the chemotherapy regimen included only the platinum-based regimen. Finally, patients with AJCC stage I–IVA OPSCC receiving definitive CCRT were enrolled into this study. From the TCRD, we identified patients who underwent ^18^F-FDG PET–CT within 0 to 90 days before the index date. Patients with a record of ^18^F-FDG PET–CT were considered to have undergone pretreatment PET–CT, whereas those without records were considered to have not undergone pretreatment PET–CT. All patients in the control group (nonpretreatment ^18^FDG PET–CT) received head and neck MRI for primary tumor and nodal staging as well as abdominal ultrasound and chest X-ray for metastatic staging. The primary outcome of interest was all-cause death, which was evaluated from the initial date to the date of death. Information on OS was obtained from the Cause of Death database. Patients whose death records could not be found were considered alive, and their data were censored on the last day of the database record (December 31, 2019). To compare their survival outcomes, these patients were categorized into two groups on the basis of pre-CCRT PET–CT: Group 1, comprising those undergoing pre-CCRT PET–CT, and Group 2, comprising those receiving non-pre-CCRT PET–CT.

### Study covariates

Comorbidities were scored using the Charlson comorbidity index (CCI).^[[[28]]]^ Only comorbidities observed 12 months before and after the index date were analyzed in this study. Comorbidities were identified according to the main International Classification of Diseases, Tenth Edition, Clinical Modification (ICD-10-CM) diagnosis code in the records for the first admission for OPSCC or more than two repeated main diagnosis codes in the records for outpatient visits. To reduce the effects of potential confounders on the comparison of the survival outcome between the pre-CCRT PET–CT and non-pre-CCRT PET–CT groups, a Cox proportional regression model was adopted. The following confounders were adjusted for in multivariable regression analysis: sex, age, AJCC clinical stage, differentiation, CCI score, diagnosis year, and hospital volume (hospitals with high or low patient volumes; high-volume hospitals were defined as the top 10% of centers by the number of patients treated from 2008 to 2018). Multivariable Cox regression analysis was performed to calculate the hazard ratio (HR) for determining whether sex, age, AJCC clinical stage, differentiation, CCI score, diagnosis year, and hospital volume were significant independent predictors. The independent predictors were controlled for in the analysis, and the endpoint was mortality in the cases and controls, with Group 1 serving as the control arm.

### Statistical analysis

The cumulative mortality rate was estimated using the Kaplan–Meier method. Differences between the pre-CCRT PET–CT and non-pre-CCRT PET–CT groups were determined using the log-rank test. After adjustment for confounders, the Cox proportional regression model was used to model the time from the index date to all-cause mortality among the cases and controls. HRs were calculated in multivariate analysis with adjustment for sex, age, AJCC clinical stage, differentiation, CCI score, diagnosis year, and hospital volume. All analyses were performed using SAS (version 9.4; SAS, Cary, NC, USA). Two-tailed *P* < 0.05 was considered statistically significant.

## Results

### Study Population

A total of 3942 patients (1663 and 2279 in the pre-CCRT PET–CT and non-pre-CCRT PET–CT groups, respectively) were recruited to this study. Table 1 summarizes the characteristics of the patients. The mean age (standard deviation) of the case and control groups was 54.8 (10.0) and 55.2 (10.5) years, respectively, and their mean follow-up duration was 52.5 and 50.2 months, respectively. The 10-year interval of age was nearly balanced between the two groups (Table 1). No significant differences in sex, age, clinical tumor (cT) stage, cumulative platinum dose, and CCI score were observed between the case and control groups. A higher proportion of the patients in the case group had advanced AJCC stages, advanced clinical nodal (cN) stages, poor differentiation, 2015 to 2018 as diagnosis years, and treatments in hospitals with high patient volumes. The median dose and fraction numbers of RT in both the groups were 70 Gy and 35 fractions, respectively. The mortality rate was 61.0% and 64.5% in the case and control groups, respectively (Table 1).

**Table 1:** Demographic characteristics of patients with newly diagnosed p16-negative oropharyngeal squamous cell carcinoma receiving concurrent chemoradiotherapy

	Total N = 3942		Pre-CCRT PET-CT N = 1663		No Pre-CCRT PET-CT N = 2279		*P* value
	n	(%)	n	(%)	n	(%)	
Sex							
Male	3633	(92.2)	1528	(91.9)	2105	(92.4)	0.5774
Female	309	(7.8)	135	(8.1)	174	(7.6)	
Age (years)							
Mean (SD)	55.0	(10.3)	54.8	(10.0)	55.2	(10.5)	0.2550
Median (Q1–Q3)	54	(48–61)	54	(47–61)	54	(48–61)	
≤ 40	230	(5.8)	88	(5.3)	142	(6.2)	0.2600
41–50	1166	(29.6)	514	(30.9)	652	(28.6)	
51–60	1517	(38.5)	637	(38.3)	880	(38.6)	
61–70	700	(17.8)	298	(17.9)	402	(17.6)	
> 70	329	(8.3)	126	(7.6)	203	(8.9)	
AJCC clinical stages							
I–II	422	(10.7)	168	(10.1)	254	(11.1)	0.0111
III–IVA	3520	(89.3)	1495	(89.9)	2025	(88.9)	
Clinical T stages							
T1–T2	1813	(46.0)	789	(47.4)	1024	(44.9)	0.1180
T3–T4	2129	(54.0)	874	(52.6)	1255	(55.1)	
Clinical N stages							
N0–N1	1251	(31.7)	475	(28.6)	776	(34.1)	0.0003
N2–N3	2691	(68.3)	1188	(71.4)	1503	(65.9)	
Differentiation							
Well	273	(4.6)	91	(5.5)	182	(8.0)	< 0.0001
Moderate	2399	(40.1)	1007	(60.6)	1392	(61.1)	
Poor	1270	(21.2)	565	(33.9)	705	(30.9)	
Platinum cumulative dose, mg							
Mean (SD)	542.4	(430.7)	533.2	(420.2)	549.6	(438.6)	0.4215
Median (Q1–Q3)	450.0	(300.0—650.0)	450.0	(300.0–650.0)	450.0	(300.0–700.0)	
CCI scores							
Mean (SD)	0.7	(1.2)	0.6	(1.2)	0.7	(1.2)	0.3871
Median (Q1–Q3)	0	(0–1)	0	(0–1)	0	(0–1)	
0	2578	(65.4)	1096	(65.9)	1482	(65.0)	0.3818
1	747	(18.9)	322	(19.4)	425	(18.6)	
2+	617	(15.7)	245	(14.7)	372	(16.3)	
Diagnosis year							
2008–2010	945	(24.0)	308	(18.5)	637	(28.0)	< 0.0001
2011–2014	1161	(29.5)	448	(26.9)	713	(31.3)	
2015–2018	1836	(46.6)	907	(54.5)	929	(40.8)	
Hospital volume							
High patient volume	2547	(64.6)	1106	(66.5)	1441	(63.2)	0.0336
Low patient volume	1395	(35.4)	557	(33.5)	838	(36.8)	
Mean follow-up time, months (SD)	40.3	(34.8)	52.5	(33.1)	50.2	(36.0)	
All-cause death		2485	(63.0)	1014	(61.0)	1471	(64.5)

*PET-CT* positron emission tomography–computed tomography, *AJCC* American Joint Committee on Cancer, *TNM* tumor node metastasis, *cT* clinical tumor stage, *cN* clinical nodal stage, *CCI* Charlson Comorbidity Index, *IQR* interquartile range; HPV, human papillomavirus, *SD* standard deviation, *CCRT* concurrent chemoradiotherapy

### Prognostic factors for OS

According to the findings of the multivariable Cox regression analysis, age > 70 years, male sex, moderate to poor differentiation, advanced AJCC stages III–IVA, CCI score ≥ 1, and treatment in hospitals with low patient volumes were significant poor independent predictors of OS (Table 2). According to the results of both univariable and multivariable Cox regression analyses, the adjusted HR (aHR; 95% confidence interval [CI]) of the pre-CCRT PET–CT group was 0.90 (0.82–1.09, *P* = 0.4427, Table 2). Moreover, for the significant independent prognostic risk factors for poor OS, the aHRs (95% CIs) were 2.41 (1.97–2.95, *P* < 0.001) for male sex, 1.23 (1.00–1.52, *P* = 0.0289) for age > 70 years, 1.04 (1.01–1.32, *P* < 0.0001) for moderate differentiation, 1.17 (1.08–1.41, *P* < 0.0001) for poor differentiation, 1.81 (1.53–2.12, *P* < 0.0001) for AJCC stage III–IVA, 1.10 (1.00–1.22, *P* = 0.4891) for a CCI score of 1, 1.47 (1.32–1.64, *P* < 0.0001) for a CCI score of ≥ 2, and 1.27 (1.17–1.38, *P* < 0.0001) for treatment in hospitals with low patient volumes in the multivariable Cox regression analysis.

**Table 2:** Cox proportional hazard regression analysis of the risk of all-cause death in patients with p16-negative oropharyngeal squamous cell carcinoma receiving concurrent chemoradiotherapy

	Univariate			Multivariate		
Variable	Crude HR	95% CI	*P* value	aHR^*^	95% CI	*P* value
Pre-CCRT PET–CT (No pre-CCRT PET–CT as reference)	0.95	(0.88–1.03)	0.1986	0.90	(0.82–1.09)	0.4427
Sex						
Female	1		< 0.0001	1		< 0.0001
Male	2.67	(2.19–3.27)		2.41	(1.97–2.95)	
Age (years)						
≤ 40	1		0.0386	1		0.0289
41–50	1.03	(0.76–1.18)		1.09	(0.76–1.18)	
51–60	1.08	(0.68–1.96)		1.14	(0.70–1.29)	
61–70	1.17	(0.73–1.25)		1.18	(0.70–1.31)	
> 70	1.52	(1.25–1.85)		1.23	(1.00–1.52)	
Differentiation						
Well	1		< 0.0001			< 0.0001
Moderate	1.02	(1.00–1.10)		1.04	(1.01–1.32)	
Poor	1.16	(1.07–1.39)		1.17	(1.08–1.41)	
AJCC clinical stages						
I–II	1		< 0.0001	1		< 0.0001
III–IVA	1.49	(1.27–1.75)		1.81	(1.53–2.12)	
CCI Scores						
0	1		< 0.0001	1		< 0.0001
1	1.14	(1.03–1.27)		1.10	(1.00–1.22)	
2+	1.63	(1.47–1.81)		1.47	(1.32–1.64)	
Diagnosis year						
2008–2010	1		0.3212	1		0.1201
2011–2014	0.96	(0.87–1.06)		0.97	(0.87–1.07)	
2015–2018	0.85	(0.77–1.04)		0.84	(0.73–1.04)	
Hospital volume						
High patient volume	1		< 0.0001	1		< 0.0001
Low patient volume	1.23	(1.13–1.33)		1.27	(1.17–1.38)	

*PET-CT* positron emission tomography–computed tomography; HR, hazard ratio, *aHR* adjusted hazard ratio, *CI* confidence interval, *AJCC* American Joint Committee on Cancer, *TNM* tumor node metastasis, *cT* clinical tumor stage, *cN* clinical nodal stage, *CCI* Charlson Comorbidity Index, *CCRT* concurrent chemoradiotherapy, *HPV* human papillomavirus

^*^All covariates mentioned in Table 2 were adjusted for

### Stratified analysis of clinical stages

The results of the multivariable Cox regression analysis revealed that age > 70 years, male sex, moderate to poor differentiation, CCI score ≥ 1, and treatment in hospitals with low patient volumes were significant poor independent predictors of OS in the patients with early stages (I–II) and advanced stages (III–IVA) of OPSCC (Table 3). Pre-CCRT PET–CT was not a significant prognostic factor for OS in the patients with early stages (I–II) of OPSCC receiving standard CCRT. In the multivariable Cox regression analysis, the aHR (95% CI) of all-cause death for advanced stage III–IVA OPSCC was 0.75 (0.87–0.94, *P* = 0.0236, Table 3) in the pre-CCRT PET–CT group compared with the non-pre-CCRT PET–CT group.

**Table 3:** Cox proportional hazard regression analysis of the risk of all-cause death in patients with oropharyngeal squamous cell carcinoma patients receiving concurrent chemoradiotherapy, stratified by the AJCC clinical stage

Variable	Stage I–II			Stage III–IVA		
	aHR*	95% CI	*P* value	aHR*	95% CI	*P* value
Pre-CCRT PET-CT (No Pre-CCRT PET-CT as reference)	1.19	(0.90–1.43)	0.1566	0.75	(0.87–0.94)	0.0236
Sex						
Female	1		< 0.0001	1		< 0.0001
Male	3.14	(1.98–4.98)		2.26	(1.80–2.82)	
Age (years)						
≤ 40	1		0.0120	1		0.0251
41–50	1.01	(0.52–1.25)		1.02	(0.75–1.10)	
51–60	1.07	(0.46–1.15)		1.05	(0.70–1.12)	
61–70	1.09	(0.47–1.27)		1.15	(0.69–1.24)	
> 70	1.16	(1.07–1.89)		1.23	(1.08–1.55)	
Differentiation						
Well	1		0.0453	1		< 0.0001
Moderate	1.01	(1.00–1.25)		1.03	(1.01–1.19)	
Poor	1.15	(1.02–1.63)		1.19	(1.13–1.44)	
AJCC clinical stages						
I	1		0.5363	–	–	
II	1.13	(0.86–1.50)		–	–	
III	–	–		1		0.2310
IV	–	–		1.10	(0.92–1.35)	
CCI scores						
0	1		< 0.0001	1		< 0.0001
1	1.32	(1.06–1.65)		1.07	(1.03–1.20)	
2 +	1.80	(1.43–2.26)		1.43	(1.27–1.62)	
Diagnosis year						
2008–2010	1		0.1471	1		0.1734
2011–2014	0.91	(0.74–1.13)		0.99	(0.88–1.11)	
2015–2018	0.72	(0.58–1.11)		0.84	(0.74–1.04)	
Hospital volume						
High patient volume	1		0.0023	1		< 0.0001
Low patient volume	1.34	(1.11–1.62)		1.27	(1.16–1.39)	

*PET-CT* positron emission tomography–computed tomography, *HR* hazard ratio, *aHR* adjusted hazard ratio, *CI* confidence interval; *AJCC* American Joint Committee on Cancer, *TNM* tumor node metastasis; *cT* clinical tumor stage, *cN* clinical nodal stage, *CCI* Charlson Comorbidity Index, *CCRT* concurrent chemoradiotherapy, *HPV* Human Papillomavirus

^*^All covariates mentioned in Table 2 were adjusted for

### Survival curves of case and control groups

**Fig. 1:**
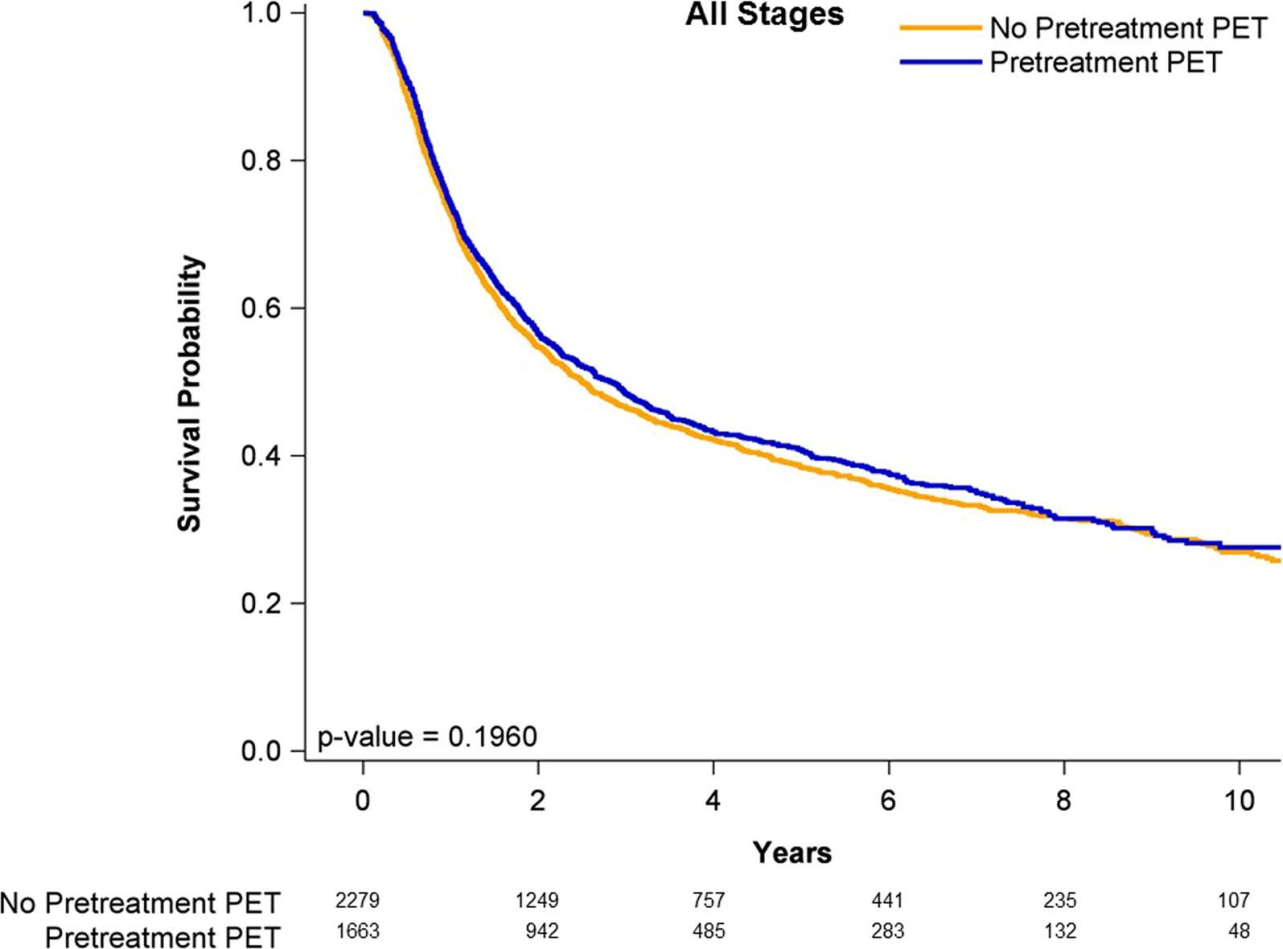
Kaplan–Meier curves of overall survival for patients with all stages of p16-negative oropharyngeal squamous cell carcinoma receiving concurrent chemoradiotherapy

**Fig. 2:**
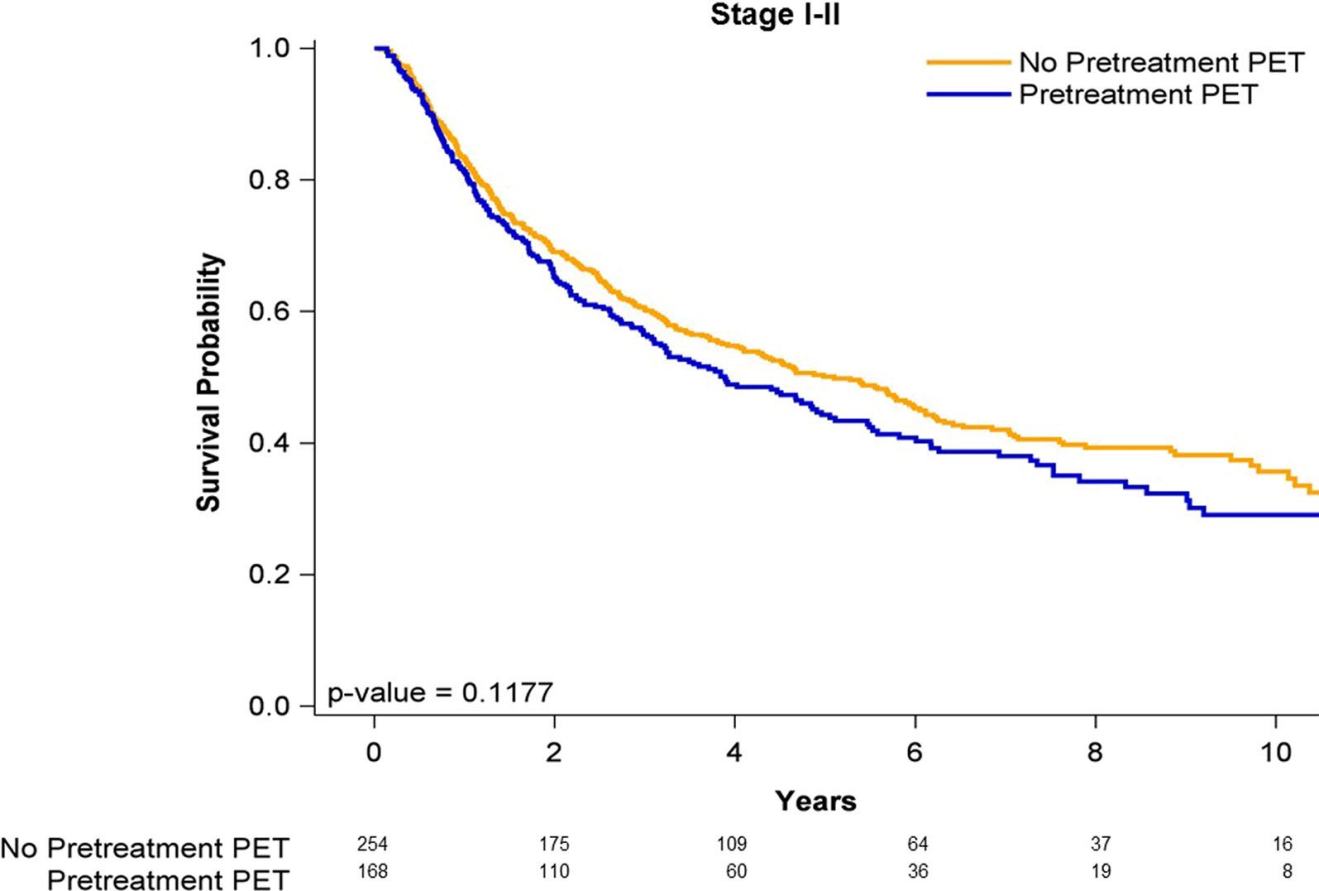
Kaplan–Meier curves of overall survival for patients with early stages of p16-negative oropharyngeal squamous cell carcinoma receiving concurrent chemoradiotherapy

**Fig. 3:**
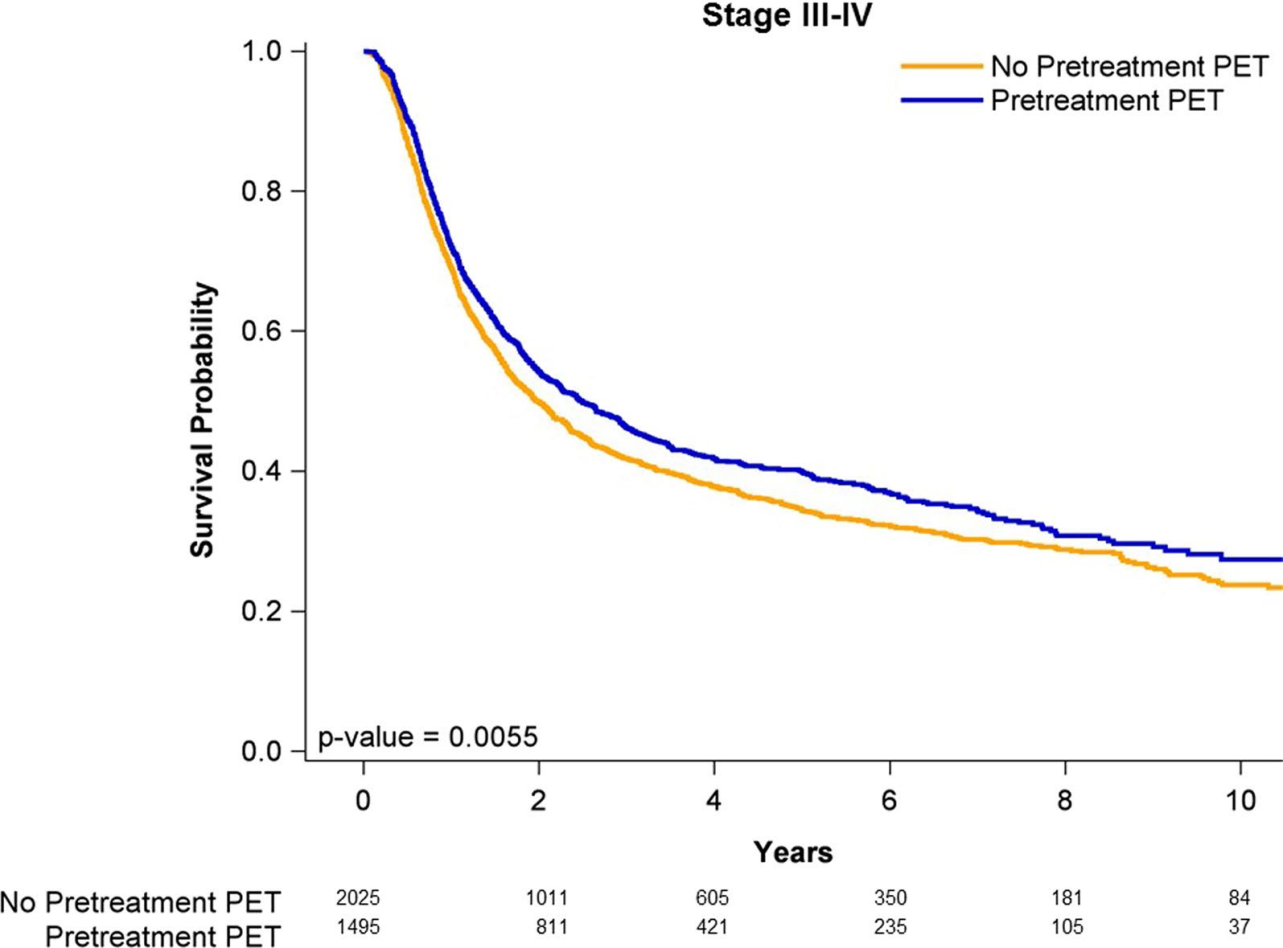
Kaplan–Meier curves of overall survival for patients with advanced stages of p16-negative oropharyngeal squamous cell carcinoma receiving concurrent chemoradiotherapy

Figure 1 shows the Kaplan–Meier curves for the OS outcomes of all stages of OPSCC in the pre-CCRT PET–CT and non-pre-CCRT PET–CT groups. The OS rate was not significantly higher in the patients undergoing either pre-CCRT PET–CT or non-pre-CCRT PET–CT (log-rank test, *P* = 0.1960). The 5-year OS rates were 43.6% and 41.1% in the pre-CCRT PET–CT and non-pre-CCRT PET–CT groups, respectively. Figure 2 presents the OS curves for the patients with stage I–II OPSCC in the pre-CCRT PET–CT and non-pre-CCRT PET–CT groups. The OS rate was not significantly higher in the patients undergoing either pre-CCRT PET–CT or non-pre-CCRT PET–CT (log-rank test, *P* = 0.1177). The 5-year OS rates were 52.1% and 50.7% in the pre-CCRT PET–CT and non-pre-CCRT PET–CT groups, respectively. Figure 3 presents the OS curves for the patients with stage III–IVA OPSCC in the pre-CCRT PET–CT and non-pre-CCRT PET–CT groups. The OS rate was significantly higher in the patients undergoing pre-CCRT PET–CT (log-rank test, *P* = 0.0055). The 5-year OS rates were 42.89% and 36.3% in the pre-CCRT PET–CT and non-pre-CCRT PET–CT groups, respectively.

## Discussion

According to the NCCN guidelines [14], early or advanced stages of p16-negative OPSCC can be treated with RT alone or CCRT. Routine use of pretreatment PET–CT for p16-negative OPSCC is still under debate. Optimal indications for pretreatment PET–CT remain unclear. In theory, accurate staging is the key step for administering appropriate treatment to patients with head and neck cancers [29]. Pre-CCRT PET–CT might be beneficial for the precise delineation of the target irradiation volume in RT [30], detection of occult metastasis and synchronous primary cancer, and determination of accurate nodal stages [13,18,19]. Furthermore, RT planning with the aid of pre-CCRT PET–CT can more precisely delineate the high radiation dose volume to reduce irradiation to normal tissues, thus resulting in few acute and chronic RT-related side effects, increasing treatment compliance, and improving survival outcomes [31–33]. The aforementioned advantages might result in the long-term survival of patients with OPSCC. However, no comparative study with an adequate sample size and long-term follow-up has examined the survival outcomes of patients with OPSCC undergoing pre-CCRT PET–CT. This is the first comparative study to evaluate the survival benefits of pre-CCRT PET–CT in patients with stage I–IVA p16-negative OPSCC.

Most previous studies have investigated both p16-positive and p16-negative OPSCC [16]. However, the survival outcomes of p16-positive and p16-negative OPSCC are different, although patients receive the same treatments [5,6]. Most patients with p16-positive OPSCC present with locoregionally advanced disease and thus have a more favorable prognosis than do those with p16-negative OPSCC [5,6]. No differences currently exist in the treatment approach, although many prospective clinical trials are investigating treatment de-escalation in HPV associated OPSCC. Therefore, in our study, we excluded patients with p16-positive OPSCC to prevent differences in survival outcomes between p16-positive and p16-negative OPSCC. In both the case and control groups, we included patients with p16-negative OPSCC.

Many studies have reported that PET–CT can be used for determining the response to treatments, including CCRT, or for the detection of recurrence in head and neck cancers [21,22]. However, few studies have evaluated whether pretreatment PET–CT is associated with improved survival in OPSCC. A recent study reported that the utilization of pretreatment ^18^F-FDG PET for the staging of nonmetastatic esophageal malignancy was associated with a lower risk of death, even after adjustment for age, stage, histology, and tumor location [34]. Routine use of pretreatment PET–CT might be unnecessary for each patient with OPSCC receiving CCRT. The present study indicated the survival benefit of pre-CCRT PET–CT in the patients with nonmetastatic p16-negative OPSCC receiving CCRT; this finding is similar to that of a previous study reporting improved survival in patients with nonmetastatic esophageal malignancy after pretreatment ^18^F-FDG PET [34]. The results of our study can be used to develop future health policies and health insurance payment standards in terms of imaging and treatment modalities for patients with OPSCC.

Compared with the control group, more patients in the case group had advanced AJCC stages, advanced cN stages, and poor differentiation, which were identified as poor prognostic factors for OS. Despite the presence of more poor prognostic factors for survival in the PET–CT group, the crude mortality rates of the pre-CCRT PET–CT and non-pre-CCRT PET–CT groups were 61.0% and 64.5%, respectively (Table 1). The utilization of pre-CCRT ^18^F-FDG PET–CT in the patients with nonmetastatic p16-negative OPSCC was associated with a lower risk of death after adjustment for sex, age, AJCC clinical stage, differentiation, CCI score, diagnosis year, and hospital volume. Because of the presence of more factors for poor OS in the PET–CT group, the survival benefit might be underestimated in this group. Thus, our study findings regarding the use of pre-CCRT PET–CT for improving the OS of patients with p16-negative OPSCC would not be overturned.

In the multivariable analysis, we observed that age > 70 years [35], male sex [36], moderate to poor differentiation [37], advanced AJCC stages III–IVA [38], CCI score ≥ 1 [39], and treatment in hospitals with low patient volumes[40] were independent poor prognostic factors for the patients with p16-negative OPSCC receiving CCRT; this finding is similar to those of previous studies[35–39] including patients with heterogeneous head and neck squamous cell carcinoma such as oral cavity cancer, OPSCC, laryngeal cancer, and hypopharyngeal cancer. This is the first study to identify independent prognostic factors for OS for patients with p16-negative OPSCC receiving CCRT. Torabi et al. reported that patients with head and neck squamous cell carcinoma who received treatment at hospitals with high patient volumes tended to have prolonged survival than did those who received treatment in hospitals with low patients volumes [40]. Although some studies have reported that patients with other head and neck cancers receiving treatment at hospitals with high patient volumes appeared to have improved survival [41–43], this is the first study to identify receiving treatment in hospitals with low patient volumes as a prognostic factor for OS in patients with p16-negative OPSCC receiving CCRT. This finding may be due to differences in clinical practice, chemotherapy delivery, and RT techniques between hospitals with low and high patient volumes [40–43].

As shown in Table 3, compared with non-pre-CCRT PET–CT, pre-CCRT PET–CT did not improve OS in the patients with stage I–II p16-negative OPSCC receiving CCRT. This finding might be attributable to the risk of occult neck metastases in the patients with early-stage (T1/T2) OPSCC and a clinically negative neck [44,45]. Thus, the elective treatment of the neck should be strongly considered [44,45]. Elective treatment of the neck can be achieved through neck irradiation. This approach is generally consistent with the guidelines of the American Society of Clinical Oncology and the NCCN [14,46]. Thus, elective neck irradiation (approximately 50 Gy) for stage I–II OPSCC is routinely used [47], irrespective of the use of PET–CT. For advanced stages of OPSCC, PET–CT can accurately detect cervical nodal or distant metastases [13,18,19] and thus prevent undertreatment with low-dose irradiation (< 70 Gy) to gross lymph node metastasis detected through PET–CT or overtreatment of OPSCC with distant metastasis. PET–CT is beneficial for detecting distant metastases, unknown primary lesions, and synchronous second primary tumors as well as for altering radiation fields and doses in patients not undergoing neck dissection [7–9]. Thus, the use of PET–CT for patients with advanced stages (III–IVA) of OPSCC receiving CCRT is beneficial for OS because it enables more accurate staging and optimal treatment, can determine accurate target volumes for RT and precisely delineate RT fields, and enables the early detection of second primary cancer with synchronous treatment [7–9,13,18,19]. PET imaging alone or in combination with CT improved the tumor–node–metastasis staging of primary cancer and altered management in 13.7% of patients [13]; thus, more accurate staging was associated with more precise treatment [10]. In addition, 18FDG-PET–CT findings can facilitate radiotherapy planning [48] by allowing the determination of precise irradiated target volume and accurate delineation of gross tumor volume to be irradiated, thereby lowering RT-related toxicity [49–51]. CCRT is the mainstay of initial treatment for patients with early and locoregionally advanced OPSCC [52]; therefore, pretreatment 18FDG-PET–CT could help with more precise RT planning and accurate staging for optimal OPSCC treatment matching [48]. This is the first study to demonstrate an association of pre-CCRT PET–CT with improved OS in patients with advanced stages (III–IVA) of OPSCC. Routine use of pre-CCRT PET–CT is not suggested for each patient with p16-negative OPSCC because PET–CT was not associated with improved OS in the patients with stage I–II p16-negative OPSCC in this study. This is the first study to demonstrate that pre-CCRT PET–CT improved OS in the patients with stage III–IVA OPSCC, but might be not in those with stage I–II OPSCC. These findings can guide physicians and patients for shared decision-making regarding undergoing expensive imaging modalities such as PET–CT.

The strength of our study is that it is the first largest homogenous modality study on PET–CT including a long-term follow-up cohort to examine the survival outcomes of pre-CCRT ^18^FDG PET–CT or non-pre-CCRT PET–CT in patients with OPSCC receiving standard CCRT stratified by different clinical stages. No comparative study has investigated the outcomes of ^18^FDG PET–CT by different clinical stages and has included a sufficient sample size and a long-term follow-up period. Pre-CCRT ^18^FDG PET–CT was associated with survival benefits only for patients with stage III–IVA p16-negative OPSCC, with no associated with the survival benefits in those with stage I–II p16-negative OPSCC. Our results suggest that pre-CCRT ^18^FDG PET–CT is unnecessary for each patient with OPSCC. Thus, we do not recommend ^18^FDG PET–CT for every patient with OPSCC. Pretreatment ^18^FDG PET–CT should be used only for patients with stage III–IVA OPSCC (Table 3 and Fig. 3). Our findings can be incorporated into national health policies to reduce unnecessary medical expenditure. Our results should be considered in future clinical practice and prospective clinical trials.

This study has some limitations. First, because all the patients with p16-negative OPSCC were enrolled from an Asian population, the corresponding ethnic susceptibility compared with that of a non-Asian population remains unclear; hence, our results should be cautiously extrapolated to non-Asian populations. However, no evidence indicates differences in the survival outcomes of patients with p16-negative OPSCC receiving CCRT between Asian and non-Asian populations. Second, the toxicity scores have not been available in the TCRD. Third, the diagnoses of all comorbidities were based on ICD-10-CM codes. However, the combination of the TCRD and National Health Insurance Research Database in Taiwan appears to be a valid resource for population research on cardiovascular disease, stroke, or chronic comobidities [53–55]. Moreover, the Taiwan Cancer Registry Administration randomly reviews charts and interviews patients to verify the accuracy of diagnoses, and hospitals with outlier chargers or practices may be audited and subsequently heavily penalized if malpractice or discrepancies are identified. To obtain crucial information on population specificity and disease occurrence, a large-scale randomized trial comparing carefully selected patients undergoing suitable treatments is essential. However, performing randomized controlled trials in daily practice might be difficult because not administering PET–CT to patients with advanced stages of OPSCC for their inclusion in the control group would be unethical. Despite these limitations, a major strength of this study is the use of a nationwide population-based registry with detailed baseline and treatment information. Lifelong follow-up was possible through the linkage of the registry with the national Cause of Death database. Considering the magnitude and statistical significance of the observed effects in the current study, the limitations are unlikely to affect our conclusions.

## Conclusions

Routine use of pre-CCRT ^18^FDG PET–CT is not necessary for every patient with p16-negative OPSCC. Pre-CCRT ^18^FDG PET–CT is associated with improved survival in patients with stage III–IVA p16-negative OSCC, but might be not in those with stage I–II p16-negative OPSCC.

## Data Availability

We used data from the National Health Insurance Research Database and Taiwan Cancer Registry database. The authors confirm that, for approved reasons, some access restrictions apply to the data underlying the findings. The data used in this study cannot be made available in the manuscript, the supplemental files, or in a public repository due to the Personal Information Protection Act executed by Taiwan’s government, starting in 2012. Requests for data can be sent as a formal proposal to obtain approval from the ethics review committee of the appropriate governmental department in Taiwan. Specifically, links regarding contact info for which data requests may be sent to are as follows: http://nhird.nhri.org.tw/en/Data_Subsets.html#S3 and http://nhis.nhri.org.tw/point.html.

## Abbreviations

PET–CT: Positron emission tomography–computed tomography
^18^-FDG PET–CT: 18-Fluorodeoxyglucose positron emission tomography–computed tomography
HR: Hazard ratio
aHR: Adjusted hazard ratio
CI: Confidence interval
FDG: Fluorodeoxyglucose
NCCN: National Comprehensive Cancer Network
AJCC: American Joint Committee on Cancer
TNM: Tumor node metastasis
cT: Clinical tumor stage
cN: Clinical nodal stage
CCI: Charlson comorbidity index
OS: Overall survival
ICD-10-CM: International classification of diseases, tenth revision, clinical modification
IQR: Interquartile range
RT: Radiotherapy
CCRT: Concurrent chemoradiotherapy
CT: Computed tomography
MRI: Magnetic resonance imaging
IMRT: Intensity-modulated radiation therapy
VMAT: Volumetric modulated arc therapy
HPV: Human papillomavirus
OPSCC: Oropharyngeal squamous cell carcinoma
TCRD: Taiwan cancer registry database
